# Role of Matrix Metalloproteinases in Musculoskeletal Diseases

**DOI:** 10.3390/biomedicines10102477

**Published:** 2022-10-04

**Authors:** Lokender Kumar, Monish Bisen, Azhar Khan, Pradeep Kumar, Sanjay Kumar Singh Patel

**Affiliations:** 1 School of Biotechnology, Faculty of Applied Sciences and Biotechnology, Shoolini University, Solan 173229, India; 2 Department of Chemical Engineering, Konkuk University, Seoul 05029, Korea

**Keywords:** musculoskeletal diseases, matrix metalloproteinase, extracellular matrix

## Abstract

Musculoskeletal disorders include rheumatoid arthritis, osteoarthritis, sarcopenia, injury, stiffness, and bone loss. The prevalence of these conditions is frequent among elderly populations with significant mobility and mortality rates. This may lead to extreme discomfort and detrimental effect on the patient’s health and socioeconomic situation. Muscles, ligaments, tendons, and soft tissue are vital for body function and movement. Matrix metalloproteinases (MMPs) are regulatory proteases involved in synthesizing, degrading, and remodeling extracellular matrix (ECM) components. By modulating ECM reconstruction, cellular migration, and differentiation, MMPs preserve myofiber integrity and homeostasis. In this review, the role of MMPs in skeletal muscle function, muscle injury and repair, skeletal muscle inflammation, and muscular dystrophy and future approaches for MMP-based therapies in musculoskeletal disorders are discussed at the cellular and molecule level.

## 1. Introduction

Diverse cellular communication networks control tissue homeostasis and the remodeling of extracellular matrix (ECM) [1]. The ECM offers structural support and aids in creating dynamic environments for cells and signal molecules. Cellular proliferation and tissue architecture are tightly regulated during healthy development and musculoskeletal diseases. MDs are a substantial contributor to the need for rehabilitation, particularly in children, and MD patients are predisposed to other diseases, such as cardiovascular disorders, and are at a higher risk for mental illnesses [2]. MD types include the common conditions related to joints (arthritis, rheumatoid arthritis, osteoarthritis (OA), psoriatic arthritis, and gout); bones (osteoporosis, traumatic fractures, and osteopenia); muscles (sarcopenia); other parts of the body (neck and back pain, fibromyalgia, vasculitis) [3] and genetic disorders such as achondroplasia, muscular dystrophy, and osteogenesis imperfecta [4]. Matrix metalloproteinases (MMPs) play a crucial role in maintaining the structural integrity of ECM [5]. Pathophysiological events in musculoskeletal diseases promote ECM remodeling and MMPs expression [1,6]. MMPs are primarily accountable for the degradation and turnover of ECM matrix proteins. In addition to MMPs, serine proteases, proteosomes, and cysteine proteases may contribute to extracellular matrix remodeling [7]. Initially, Couch and Strittmatter investigated the role of metalloproteinase in skeletal muscle cell fusion [8], and later Guérin and Holland conducted detailed studies on MMPs expression in human muscle cells [9]. Further, the role of MMPs in myogenic cell migration and skeletal muscle plasticity has been the focus of research [10,11]. The role of MMPs and tissue inhibitors of metalloproteinases (TIMPs) in injury, regeneration, and skeletal muscle function has been explored [12]. TIMPs were initially identified as MMP inhibitors, but their significance has expanded to include cell differentiation, migration, angiogenesis, synaptic plasticity, and apoptosis [13]. Molecular understanding of the TIMP-MMP complex has shown many binding sites that can selectively inhibit metalloprotease activity [14]. As cell-matrix and cell–cell interactions alter the tissue environment, MMPs may play a key role in tissue homeostasis and regeneration [10,15]. MMPs and TIMPs expression must be tightly regulated to maintain a typical cellular environment, and any dysregulation might result in severe pathological disorders [15].

Tissue damage induces a transient interference in the protease balance at the molecular and cellular levels, which may resolve following tissue regeneration. In chronic diseased conditions, however, the protease network imbalance may directly influence the critical regulatory system, resulting in a persistent malfunction of the complex cellular signaling network [16]. MMPs have a broad spectrum of substrates, including intracellular proteins, secreted proteins, membrane-bound proteins, and extracellular matrix proteins [17]. According to animal model studies using gene knock-out strategy, it was shown that the MMPs have a multifaceted function in physiology [18,19,20]. Therefore, MMPs, despite their complicated role and capabilities, have been an attractive target for developing therapeutics for extracellular tissue remodeling in skeletal muscle diseases [8,12]. This review focuses on the significance of MMPs in skeletal muscle function, injury, repair, and inflammation at the molecular, cellular, tissue, and organ levels.

## 2. Extracellular Matrix (ECM) Components

**Figure 1:**
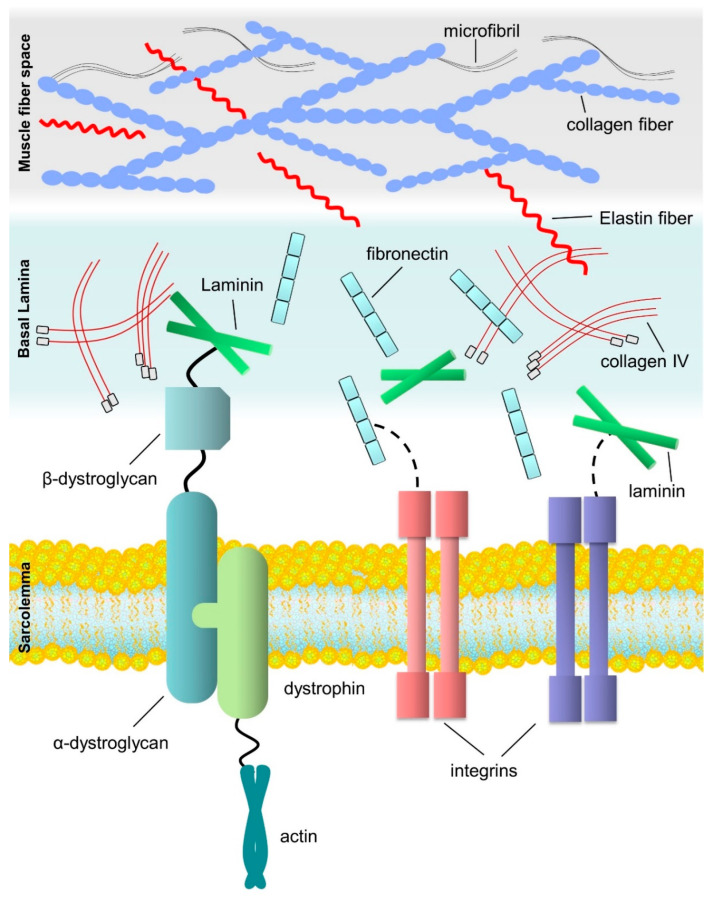
Schematic representation of the extracellular matrix environment (muscle fiber space), basal lamina, and sarcolemma of skeletal muscle.

The ECM is a network of complex macromolecules, including structural proteins, proteins involved in processing the matrix, and related proteins (Figure 1). The ECM promotes the structural integrity of the tissue and organs by providing structural and functional support. The ECM contains fibers, proteoglycans, glycoproteins, and polysaccharides. The ECM proteins regulate cell movements, differentiation, growth, and development. The fiber network of ECM contains macromolecular proteins such as collagen, laminin, elastin, and fibronectin. Collagen is an abundant component in the ECM and provides structural support to cells. The tissue can be divided into bones (harder) and cartilage (softer) based on the mineralization intensity. Various forms of collagen are mentioned in Table 1 [21]. The collagen protein consists of three alpha chains (α1, α2, and α3) in triple helix symmetry, and it has a high glycine content to stabilize the helical structure and facilitate the cross-link formation. The basal lamina of muscle fiber contains laminin that promotes the activation and expression of integrins. The deficiency of laminin may lead to abnormalities in the ECM architecture and function [22]. Laminin-1 maintains the attachment of muscle fiber with the basal lamina, is responsible for quick regeneration, and increases myoblast cell migration. Mutations in laminin may lead to muscle fiber dissociation from the basal lamina, causing atrophy and abnormal muscle-fiber development [23]. Fibroblasts secrete fibronectin that is responsible for activating integrin proteins instigating peripheral nuclear localization. Mutations in fibronectin can cause ECM structural and functional abnormalities [24]. Fibronectin can inhibit myoblasts’ migration and promote the differentiation and attachment of myoblasts. Fibronectin deficiency can lead to ECM structural and functional abnormalities [25]. Dystroglycan and dystrophin proteins are essential for the interaction between the cytoskeleton and ECM [26]. Dystrophin binds with actin protein via actin-binding domain; this interaction is helpful in maintaining the structural integrity of the cell membrane. Inhibition of dystroglycan may cause cytoskeleton abnormalities and increase susceptibility to contractile damage [27]. In addition, proteoglycan is one of the critical components of skeletal muscle ECM and is involved in the connection of ECM components and the cytoskeleton. Defects in proteoglycan may lead to muscular dystrophy and muscle degeneration [28]. ECM degradation is essential for development, tissue remodeling, wound healing, inflammation, tissue injury, and morphogenesis. It is the unregulated ECM degradation that leads to the severe metabolic diseases, including musculoskeletal conditions, cancer, immune-related diseases, and neurological and cardiovascular disorders [7].

## 3. Matrix Metalloproteinases (MMPs)

MMPs belong to metzincs, a large family of zinc (Zn)-associated multidomain proteinases involved in ECM remodeling. MMPs are membrane-bound and secreted proteins that belong to the Zn-dependent endopeptidase, metazincin superfamily [29,30]. In 1949, MMPs were first described as depolymerases that facilitate tumor growth by increasing the fluidity of connective tissue. Later, the collagenase was characterized by Gross and LaPierre, which is the substance involved in tadpole tails’ resorption. Further, plenty of research has been completed on the MMPs, and researchers have identified MMPs in plants, nematodes, bacteria, viruses, and animals. Secreted MMPs are a wide subfamily of MMPs that are expressed as inactive enzymes (zymogens or pro-MMPs) [21]. Proteolytic agents such as other activated MMPs, pro-hormone convertase, furin, and plasminogen activator may activate these inactive isoforms [31]. In addition, these may also activate non-proteolytic agents such as mercurial compounds, protein desaturating agents, and reactive oxygen species [32]. In contrast, membrane-bound MMPs have a distinct transmembrane domain and a C-terminal cytoplasmic tail [21].

MMPs have been well acknowledged as biomarkers in diagnosis, disease progression, immune disorders, and treatment efficacy. For example, Huang et al. have linked MMP-9 as a potential biomarker for certain cancers (breast, pancreatic, cervical, ovarian, pancreatic, and giant cell tumor of bone) [20]. Hence, these enzymes can be recognized as an indicator of disease onset and progression. In addition, MMP-1 and MMP-3 have been associated with atherosclerosis [33]. The primary role of MMPs is the degradation and processing of ECM, matrix proteins, glycoproteins, cytokines, growth factors, and membrane receptors [12,17].

### Classes and Structural Features of MMPs

**Figure 2:**
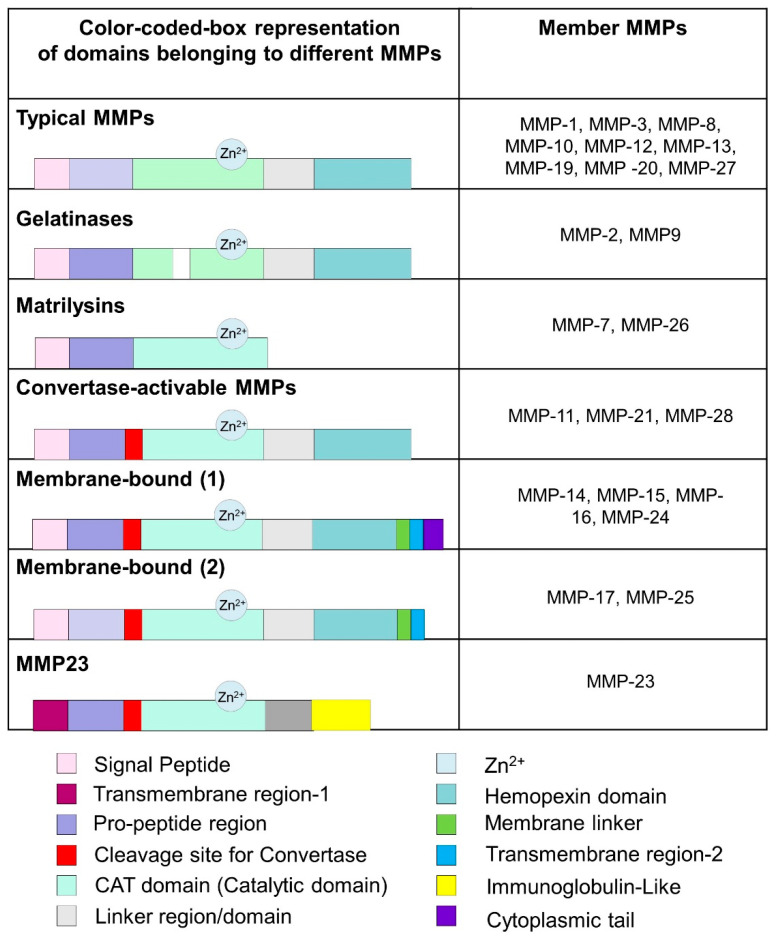
Schematic representation of the various domains of MMPs.

MMPs are classified in various groups, have substrate ranges depending upon their activity, and share similar structural and functional features. There are a minimum of 28 groups of MMPs, of which 23 are known to be expressed in humans. Based on the structural bioinformatics’ analysis, they are divided into typical or non-furin-regulated MMPs, gelatinases, matrilysins, convertase-activable MMPs, membrane-bound MMPs, and MMP-23 (Figure 2). MMPs have been classified by their features, such as substrate specificity or structural characteristics. For example, collagenases can break down fibrillar collagen types producing 1/4 C-terminal and 3/4 N-terminal fragments. This process requires unwinding the collagen chains and further hydrolyzing the peptide bonds.

**Figure 3:**
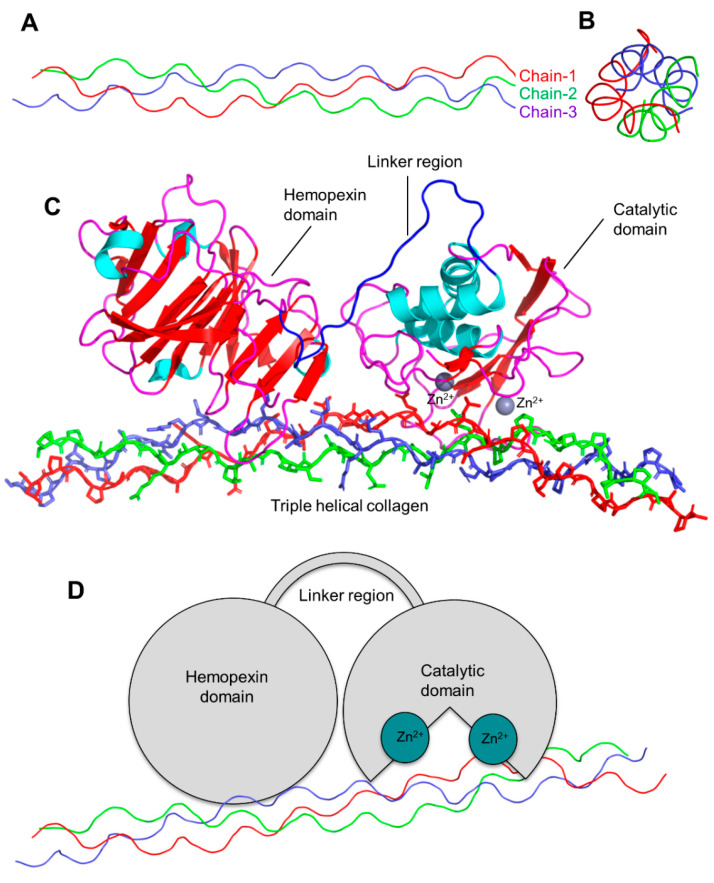
The 3D structure of collagen type-1, three chains are highlighted in red, blue, and green (triple-helical), side view (**A**), front view (**B**), 3D representation of MMP-1 (cartoon view) interacting with triple helical collagen (domain representation) (**C**), domain schematic representation of the MMP-1 interacting with collagen (**D**).

The active site of the MMPs proteinases is Zn-dependent and highly conserved. As described in Figure 3, MMP-1 has three district regions: (1) catalytic domain (containing Zn); (2) linker region; and (3) hemopexin domain. The catalytic domain interacts with triple helical collagen with Zn at the active site, enabling the degradation of collagen chains. The structural features of MMPs include specific signal features, a signal peptide (N-terminal) that is essential for the extracellular secretion of the protein (Figure 3). The pro-domain (approx. 80 amino acids long) is responsible for maintaining the inactive state of the enzyme. The catalytic domain (approx. 160 amino acids) is responsible for the activity and consists of three-helix and five beta sheets with three calcium and two Zn ions. A flexible and variable linker region (14–60 amino acids) connects the catalytic domain with the hemopexin domain. Four propeller structures characterize the hemopexin-like domain (approx. 210 amino acids). Additional features include (but are not limited to) a transmembrane domain with cytoplasmic domains (C-terminal) present in MMP-16, MMP-24, MMP-14, and MMP-15,

## 4. Function of MMPs in Musculoskeletal Diseases

MMPs are regulated on multiple levels, including (1) gene regulation, (2) pro-MMPs activation, and (3) suppression by specific inhibitors (TIMPs) by cytokines, growth factors, phorbol esters, hormonal stress, bacterial endotoxins, neoplastic transformation, and ECM remodeling events [34,35]. Transcription of MMPs is also controlled by functional cis-acting elements [36]. On the other hand, MMPs are inhibited by silencing signaling pathways that result in the inactivation of target proteins, the suppression of transcriptional activators, or the inhibition of their phosphorylation processes. MMPs regulation is primarily controlled by post-translational regulatory mechanisms and protein modifications [37]. The extensive coverage of ECM substrates provides insights into the multifaceted function of MMPs in tissue homeostasis and development [38]. In addition, tumor growth and cell invasion are promoted by the coordinated communication of MMPs with serine proteases, cysteine proteases, and urokinase plasminogen activators [39]. MMPs are also found in intercellular compartments, including nuclei and mitochondria [40]. The expression in these sites was linked with oxidative stress under various pathophysiological conditions. Further, oxidative stress may lead to increased MMP-2 expression linked with apoptotic pathways in response to tissue injury [40,41]. This has provided an avenue for exploring MMPs inhibitors as therapeutic interventions for wound healing.

**Figure 4:**
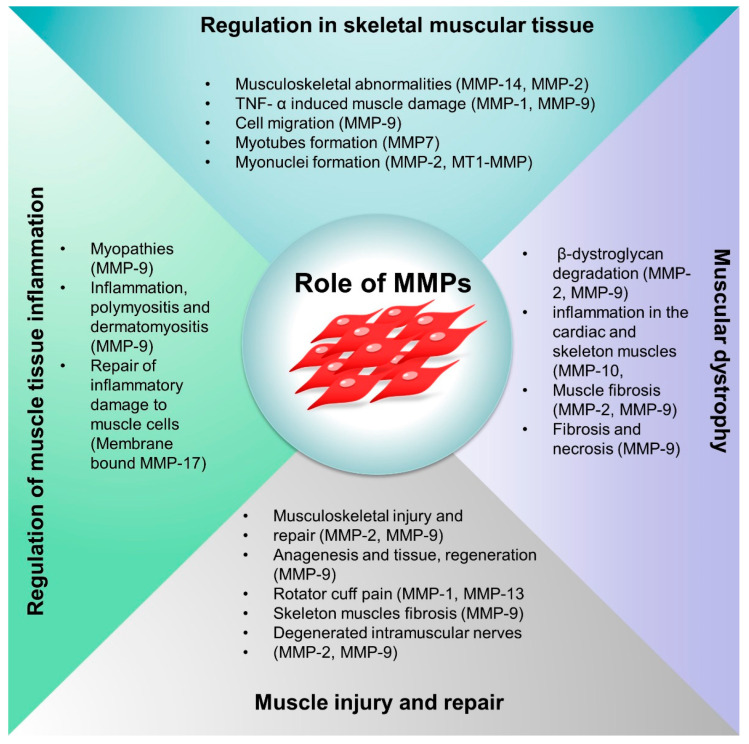
Schematic representation of the roles of MMPs in skeletal muscle regulation, muscle injury and repair, inflammation and oxidative stress, and muscular dystrophy.

MMPs knock-out mouse model studies revealed that the MMPs show subtle abnormalities in the physiological functions suggesting functional redundancy of MMPs [42,43,44]. MMP-14 deficiency resulted in skeletal muscle defects, arthritis, craniofacial dysmorphism, defects in connective tissue, defective maturation of muscle development, and decreased angiogenesis [35,45]. In addition, double MMP-2 and MMP-14 knock-out animals (mice) exhibited mortality with musculoskeletal abnormalities [46]. Depending on the stage of the disease and its effect on the protease expression network, MMPs may play a protective, aggressive, or dual role in various pathophysiological conditions. Additionally, MMPs may contribute to cell recruitment, migration, differentiation, and adhesion [47,48]. The expression of cytokines that initiate the recruitment and migration of stem cells at the site of damage is linked with the upregulation of MMPs [49]. MMPs substantially contribute to immune signaling and inflammatory processes by controlling inflammation, immune cell invasion, and the chemokine gradient in inflamed regions on the cellular and molecular levels [50]. The proteolytic processing of chemotactic fragments, cytokine receptors, and chemokines subtly influences the soft tissue inflammation. Critical limb ischemia (CLI) is characterized by skeletal muscle degeneration and decreased vascularization. Increased MMP-2 has been linked with ECM degradation, and Dang et al. showed that the MMP-2 inhibitor CTTHWGFTLC (CTT)-based nano gels have the potential to regenerate ischemic limbs via promoting endothelial cell migration [38]. We have summarized the role of MMPs in various musculoskeletal systems in Table 2 and Figure 4.

### 4.1. Role of MMPs in the Musculoskeletal System

Previously, research using rat models showed that membrane-bound and soluble MMPs played a crucial role in myoblast fusion by enhancing cell migration and fusion [76]. Gradually, the investigation expanded to comprehend the function of MMPs as a therapeutic target for musculoskeletal disorders [77], including muscular dystrophy [11]. However, the minimal success with the research called for further research in this domain [78]. The primary rationale was the complicated and multifactorial impact of MMPs on complex cellular processes, cell migration, ECM remodeling, and inflammation. This necessitates a comprehensive examination to design targeted therapeutic interventions. Muscle growth depends on the essential fibroblast growth factor (bFGF) that controls the differentiation and proliferation of fiber cells. MMPs expression was detected in the skeletal muscle of rats and linked with muscle regeneration and recovery [79]. Furthermore, MMPs facilitated the ECM remodeling that plays an essential role in the muscle cell–macrophage axis-mediated collagen turnover. Exercise stimulation and single-cell RNA sequencing showed that the macrophages in the muscle cells are the primary sources of MMP-14 [65]. This provides a valuable insight into the role of macrophage-induced muscle fiber behaviors and ECM remodeling during mechanical loading.

### 4.2. MMPs Regulation in Skeletal Muscle Tissue

Several MMPs, including MMP-1, MMP-9, MMP-7, MMP-3, MMP-2, and MMP-14, have been expressed and regulated in myogenic cells of various organisms [53,80,81]. MMP-2 expression has been well-documented in the literature; however, the role of MMP-9 is still controversial. Reports suggest that TNF-α induces MMP-1 and MMP-9, phorbol ester, and muscle tissue-damaging factors [47,52]. However, myoblasts of limb muscles show a differential expression of MMP-1 and MMP-9 [53]. Hence, these may represent a biomarker signature. In addition, MMPs expression is also regulated by chemokine and cytokine phenotypes. Mouse myoblasts showed up to a 30-fold increase in MMP-9 expression in the presence of TNF-α [54] and b-FGF; however, there was no effect on expression in the presence of IGF-I, PDGF-BB, or TGF-β [82]. The exact role of MMPs and their tissue inhibitors during myogenesis is still a mystery. Lewis et al. proposed that MMP-9 expression led to cellular migration, and TIMP-1 upregulation suppressed the cell moment, ultimately leading to cell fusion [55]. Studies involving understanding the role of MMPs in myogenesis may direct their contributions toward cell migration and cell fusion. Additional studies are required to investigate the detailed molecular mechanism of MMPs in myogenic cell fusion and migration. MMPs are involved in myotubes’ formation, and cells expressing higher levels of MMPs showed high myogenic cell fusion. MMP-7-expressing myoblasts cells showed a high tendency toward myotubes formation compared to controls, and matrilysins-expressing myoblasts showed high fiber formation [56,57]. Co-transfection of C2C12 cells with MMP-2/MT1-MMPs showed high myonuclei formation [35,58]. MMPs/TIMPs coordination has been important in myogenic cell migration. When cultured with a specific MMPs inhibitor (hydroxamic acid), mandibular explants showed an alteration in mandibular morphogenesis [83]. In addition, broad-spectrum MMPs inhibitors such as BB94 [84], GM6001 [85], and MMPs inhibitor II showed reduced cell migration in vitro [86].

### 4.3. MMPs in Musculoskeletal Injury and Repair Mechanism

The type, nature, extent, and intensity of skeletal muscle injury govern the molecular and structural alterations required for the healing and repair process. MMP-2 and MMP-9 have been associated with musculoskeletal disorders and tissue repair mechanisms in multiple animal models [59]. Cardiotoxin-induced muscle damage was associated with sustained MMP-2 and MMP-9 production [10]. Monocyte chemoattractant protein (MCP-1) and MMP-9 are responsible for the anagenesis and tissue regeneration by C2C12 cells [60]. In addition, MMP-2 regulation is linked with myotubes regeneration and maturation. MMPs are involved in the healing and degradation of tendons. Studies showed MMPs inhibitors as potential therapeutic options in tendinopathy [87]. As previous studies have demonstrated the significance of MMP-2 and MMP-9 in muscle healing, researchers also investigated the effect of long-term metal implants on animals using these proteins [88].

MMPs may upregulate cytokines and chemokines, contributing to inflammation and tendon atrophy. Blood, tissue, and glenohumeral joint fluid samples from patients with rotator cuff pain demonstrated the presence of MMP-1 and MMP-13 [61]. Aging is a crucial factor in injury recovery, and aged muscles show progressing muscle mass, strength, and quality. Chen et al. observed that the aged muscles undergo structural changes that lead to restricted ECM remodeling [89]. This was linked with a decreased expression of MMPs, TIMPs, transforming growth factor β, and cathepsins.

Further, it was shown that the muscle injury elevated the ECM degradation and decreased ECM decomposition providing significant clues regarding the use of MMPs and related proteins in age-related muscle fibrosis. A high expression of MMP-9 has also been linked to skeletal muscle fibrosis [62]. A study showed the therapeutic role of polyethylene glycol hydrogel and fibrin glue that reduced MMP-9 activity, causing a decrease in collagen density in denervated muscle fibrosis [90]. In normal animals’ (mice) muscles, MMP-9 and MMP-2 are localized at neuromuscular junctions. MMP-9 increases in the degenerated intramuscular neurons in diseased animals, and MMP-2 persists at the intersection [52]. Due to the significance of TIMP-1, MMP-9, and MMP-2 in the wound repair process, these proteins were investigated as potential biomarkers for wound age estimation. The results showed that the MMP-9, MMP-2, and TIMP-1 might not be linked with determining wound age; however, TIMP-1 could be used to differentiate vital- and postmortem-inflicted wounds [91]. The data suggest a complex regulatory framework of MMPs and their inhibitor proteins in wound healing processes. This suggests that the regulation and site-dependent expression of MMPs are also highly essential in the repair mechanisms of degenerated intramuscular nerve injury.

### 4.4. Role of MMPs in the Regulation of Inflammation in Muscle Diseases

MMPs have been linked with a critical regulator of inflammation and innate immune-response regulators [92]; therefore, MMPs are being explored as a potential target as an anti-inflammatory therapy for the treatment of muscle diseases. Many MMPs are linked with inflammatory conditions in various muscle-related disorders [73,93]. The expression, activation, and regulation of MMP-9 have been linked to inflammatory myopathies [63]. High MMP-1 and MMP-9 expression was detected in myositis, polymyositis, and dermatomyositis patients [64], and MMP-9 antibodies are found in nearly all inflammatory myopathies. MMP-2 also showed a similar pattern with low intensity compared to MMP-9. MMP-1 has shown enhanced expression and immunolocalization in diseased muscle fibers and fibroblasts [64]. In addition, overexpression of MMP-9, and MMP-1 was detected in dermatomyositis and polymyositis. MMP-14 has been linked to the ECM remodeling at the interface of macrophages, and muscle cells after an exercise incentive in mice [65]. Vascular tissue remodeling was also associated with MMPs; a study showed that the MMP-2 and MMP-9 contribute to the vascular smooth muscle fiber integrity in a rat model of cardiometabolic disease [66].

Additionally, resistance training (RT) has been linked with therapeutic effects via regulating the expression of MMPs. Vasilceac et al. showed that RT induced MMP-2 downregulation in the quadriceps tendon of rats [67]. Membrane-bound MMP-17 (exclusively expressed by muscle cells) is required to repair inflammation-induced damaged smooth muscle cells [68]. Many diseases, such as OA, involve dysregulation in ECM degradation [67]. Hence, exploring the role of therapies such as RT in MMPs expression and ECM degradation may provide insights for managing OA.

### 4.5. MMPs in Muscular Dystrophy (MD)

MDs are a set of inherited disorders characterized by gradual muscular deterioration and disintegration [94]. Muscular dystrophy is characterized by progressive muscle weakness associated with abnormalities in the interaction between the muscle cell membrane and the ECM [95]. Dystroglycan is a crucial cell-membrane receptor that is involved in cell adhesion. This also led to the hypothesis that MMPs play an essential role in the pathogenesis of the MD, muscular dystrophy. The dystroglycan complex is made up of α-dystroglycan and β-dystroglycan (43 kDa). In vitro studies indicate that MMP-2 and MMP-9 cannot halt the cleavage of β-dystroglycan, suggesting the engagement of other MMPs in the degradation process [69]. Further, in vivo studies showed that the tissue inhibitors of metalloproteinases (TIMPs) expression were elevated, causing collagen accumulation and cross-linking in Duchenne muscular dystrophy (DMD) [70]. DMD is a genetic disease linked with gradual muscle weakening, cardiomyopathy, and respiratory collapse. Baraibar-Churio et al. showed that the MMP-10 deficiency causes chronic inflammation in the cardiac and skeletal muscles in aged mdx mice [71]; thus, suggesting it as a critical therapeutic targe for muscular dystrophy. MMP-2 activity is enhanced in the muscles [96], and TIMP-1 is increased in the serum and plasma of muscular dystrophy patients [97]. MMP-10 deficiency has been linked with the progression of severe muscular dystrophy in aged dystrophic mice [71]. Muscle inflammation and oxidative stress are hallmarks of Duchenne muscular dystrophy, and the immuno-expression analysis of MMP-2 and MMP-9 revealed that these proteins are involved in muscle fibrosis in mdx animals [72]. Further, reduction in MMP-9 expression by omega-3 has contributed to muscle regeneration in a mouse model (mdx) of DMD [73]. MMPs profiling has been proposed as a practical, valuable biomarker of muscle damage in patients with muscular dystrophy. In addition, MMPs inhibition has also been explored as a potential target for therapeutic strategies against musculoskeletal disorders. Reports suggested the involvement of MMP-9 in dystrophinopathy, and MMP-9 inhibition has beneficial effects in terms of reduced fibrosis, macrophage infiltration, and decreased necrosis [62,74]. However, results also showed that the constitutive expression of MMP-9 induced no detrimental effect on muscle structure [98], and in this situation, damaging effects of MMP-9 were expected. Another study of MD patients showed that MMP-9 levels are not indicative of treatment response in DMD [75]. Such results show the complexity of MMPs regulation under various clinical scenarios. The failure of inhibition could be the inhibition of the target and related MMPs leading to an undesired effect on the cellular and tissue level.

## 5. Conclusions and Future Directions

Concerning the regulation of skeletal muscle function, the role of MMPs is poorly understood. Our knowledge is limited to MMP-1, MMP-7, and MMP-9, and we know considerably less about the additional MMPs, particularly membrane-type MMPs and TIMPs. MMPs may control skeletal muscle, injury, repair, inflammation, and chemokine activity. In the scientific literature, MMPs knock-out experiments shed light on the potential redundancy in the function of MMPs under pathophysiological situations. Understanding the intricate network of MMPs in musculoskeletal illnesses is crucial for discovering biomarkers and therapeutic targets. MMPs are essential for muscle injury-recovery pathways. MMPs are implicated in muscle fiber regeneration and tissue inflammation, and a reduction in MMPs activity may contribute to fibrosis suppression. Targeting MMP activity with a target-specific strategy and regulating MMPs function may be able to halt the progression of the disease. This can be accomplished by manipulating the MMPs function expression profile. In addition, the overexpression of MMPs (MMP-1, MMP-2, MMP-7, and MMP-9) is associated with muscle regeneration and enhanced stem-cell efficiency.

Nonetheless, further verification is still necessary. Indications were also found regarding using MMPs and TIMPs as biomarkers for patients with MD, DMD, Emery–Dreifuss muscular dystrophy, and ALS. In this regard, additional information is necessary, including a detailed investigation of MMPs expression in various myopathies and musculoskeletal illnesses. Our understanding of MMPs and their regulation in musculoskeletal disorders remains in its infancy. The focus of cell biologists has always remained within the cell. To develop novel and effective therapeutic approaches, we must also explore the extracellular interactions and the relationship between the intracellular (cytoplasmic and nuclear) and extracellular environment.

## Figures and Tables

**Table 1:** Collagen types and their classes.

Collagen Types	Classes
Fibrillar collagen	Type-I, -II, -III, -V, and -XI
MACIT- membrane-associated collagens	Type-XIII and -XVII
Basement membrane collagen	Type-IV
Facit-fibril	Type-IX, -XII, -XIV, -XIX, and -XXI
Short chain collagen	Type-VIII and -X
Multiplexin collagen	Type-XV and -XVIII
Other types	Type-VI, -VII, and -VIII

**Table 2:** Contribution of the MMPs in the muscle function/muscle diseases.

MMPs	Condition/Model	Role/Function	References
**Regulation in skeletal muscular tissue**			
MMP-14	Craniofacial dysmorphism, decreased angiogenesis	Significant abnormalities of skeletal muscles growth and function	[35,45]
MMP-2 and MMP-14	Double knock-out mice	Musculoskeletal abnormalities	[46]
MMP-1 and MMP-9	Muscle damage	Induced by TNF- α, phorbol ester, and muscle tissue damaging factors	[51,52]
MMP-1 and MMP-9	Myoblasts of limb muscles	Differential expression (biomarker signature)	[53]
MMP-9	Mouse myoblasts	30-fold increase in MMP-9 expression with TNF-α	[54]
MMP-9	Cell migration	Enhanced cell migration	[55]
TIMP-1	Cell fusion	Reduced cell migration	
MMP-7	Myotubes formation	Regulates tendency toward myotubes formation	[56,57]
MMP-2 and MT1-MMP	Co-transfection of C2C12 cells MMP-2/MT1-MMP	Myonuclei formation	[35,58]
**Muscle injury and repair**			
MMP-2 and MMP-9	Muscle injury repair in animal models	Musculoskeletal injury and repair process	[59]
MMP-2 and MMP-9	Cardiotoxin-induced injury	Prolonged expression during muscle destruction	[10]
MMP-9	Cellular model (C2C12 cells)	Anagenesis and tissue regeneration	[60]
MMP-1 and MMP-13	Rotator cuff pain (Human patients)	Increased MMP-1 and MMP-13 expression	[61]
MMP-9	Skeletal muscle fibrosis	High expression of MMPs during muscle fibrosis	[62]
MMP-2, MMP-9	Degenerated intramuscular nerves	MMP-9 (degenerated nerves) MMP-2 (neuromuscular junctions junctions)	[52]
TIMP-1	Postmortem-inflicted wounds	Biomarker for wound age	
**Regulation of muscle tissue inflammation**			
MMP-9	Myopathies	MMP-9 regulates inflammation	[63]
MMP-1 and MMP-9	Myositis, polymyositis, and dermatomyositis patients	High expression of MMP-1 and MMP-9 linked with inflammation	[64]
MMP-1, MMP-9	Polymyositis and Dermatomyositis	Overexpression of MMPs	[64]
MMP-14	Exercise incentive in mice	ECM remodeling at the interface of macrophages and muscle cell	[65]
MMP-2, MMP-9	Rat model of Cardiometabolic disease	Vascular smooth muscle fiber integrity	[66]
MMP-2	Resistance training (RT)	MMP-2 downregulation in quadriceps tendon of rats	[67]
Membrane-bound MMP-17	Repair of inflammatory damage to muscle cells	inflammation-induced damaged smooth muscle cells	[68]
**Muscular dystrophy**			
MMP-2, MMP-9	Muscular dystrophy	Unable to prevent cleavage of β-dystroglycan degradation	[69]
Tissue inhibitors of metalloproteinases (TIMPs)	Duchenne muscular dystrophy	High expression of TIMPs in vivo	[70]
MMP-10	Aged dystrophic mice	The deficiency of MMP-10 caused inflammation in the cardiac and skeletal muscles	[71]
MMP-2 and MMP-9	Muscle fibrosis	Muscle inflammation and oxidative stress	[72]
MMP-9	Mouse model (mdx) of DMD	Reduction in MMP-9 expression by omega-3 caused muscle regeneration	[73]
MMP-9	Dystrophinopathy	Inhibition of MMP-9 led to reduced fibrosis, macrophage infiltration, and decreased necrosis	[62,74]
MMP-9	MD patients	MMP-9 levels are not indicative of treatment response in DMD	[75]

## Data Availability

Data sharing not applicable.
